# Supplementary material for: Global organic and inorganic aerosol hygroscopicity and its effect on radiative forcing
Source: Nat Commun. 2023 Oct 2;14:6139. doi: 10.1038/s41467-023-41695-8 (PMC10545666; doi:10.1038/s41467-023-41695-8)
Supplement: Supplementary file 1 — Supplementary Information [file 41467_2023_41695_MOESM1_ESM.pdf]

Supplement of

Global organic and inorganic aerosol hygroscopicity and its effect on radiative forcing

Mira L. Pöhlker[1,2,3,*], Christopher Pöhlker[1], Johannes Quaas[2], Johannes Mülmenstädt[2,a], Andrea Pozzer[4,5], Meinrat O. Andreae[6,7], Paulo Artaxo[8], Karoline Block[2], Hugh Coe[9], Barbara Ervens[10], Peter Gallimore[9], Cassandra J. Gaston[11], Sachin S. Gunthe[12,13], Silvia Henning[3], Hartmut Herrmann[14], Ovid O. Krüger[1], Gordon McFiggans[9], Laurent Poulain[14], Subha S. Raj[1,12], Ernesto Reyes-Villegas[9,b], Haley M. Royer[11], David Walter[1,15], Yuan Wang[3,16], Ulrich Pöschl[1]

[1] Multiphase Chemistry Department, Max Planck Institute for Chemistry, 55128 Mainz, Germany

[2] Faculty of Physics and Earth Sciences, Leipzig Institute for Meteorology, University Leipzig, 04103 Leipzig, Germany

[3] Atmospheric Microphysics Department, Leibniz Institute for Tropospheric Research, 04318 Leipzig, Germany

[4] Atmospheric Chemistry Department, Max Planck Institute for Chemistry, 55128 Mainz, Germany

[5] Climate and Atmosphere Research Center, The Cyprus Institute, 2121 Nicosia, Cyprus

[6] Biogeochemistry Department, Max Planck Institute for Chemistry, 55128 Mainz, Germany

[7] Scripps Institution of Oceanography, University of California San Diego, La Jolla, CA 92037, USA

[8] Instituto de Física, Universidade de São Paulo, São Paulo, Brazil

[9] Department of Earth and Environmental Sciences, School of Natural Sciences, University of Manchester, Manchester, UK

[10] Université Clermont Auvergne, CNRS, Institut de Chimie de Clermont-Ferrand, 63000 Clermont-Ferrand, France

[11] Department of Atmospheric Sciences, Rosenstiel School of Marine and Atmospheric Science, University of Miami, Miami, FL 33149-1031, USA

[12] Environmental Engineering Division, Department of Civil Engineering, Indian Institute of Technology Madras, Chennai, India

[13] Center for Atmospheric and Climate Sciences, Indian Institute of Technology Madras, Chennai, India

[14] Atmospheric Chemistry Department, Leibniz-Institute for Tropospheric Research, 04318 Leipzig, Germany

[15] Climate Geochemistry Department, Max Planck Institute for Chemistry, 55128 Mainz, Germany

[16] Collaborative Innovation Center for Western Ecological Safety, Lanzhou University, 730000 Lanzhou, China

[a] now at: Pacific Northwest National Laboratory, Richland, WA 99354, USA

[b] now at: School of Engineering and Sciences, Tecnologico de Monterrey, Guadalajara, 45201, Mexico

Corresponding author: Mira L. Pöhlker (poehlker@tropos.de)

Supplementary Materials

Table S1 – S5

Fig S1 - S4

Table S1. Published retrievals of κ_{org} and κ_{inorg} based on ambient aerosol measurements from previous studies. For the optimized retrieval of κ_{org} and κ_{inorg} in this study (see Fig. 1B), the data sets from the following measurements/campaigns were used (i) full year measurement at the Amazon Tall Tower Observatory according to Pöhlker *et al.* (1), subsequently abbreviated as ATTO, (ii) PRIDE-PRD2006 measurements from Rose *et al.* (2), subsequently abbreviated as PRD, (iii) CAREBeijing-2006 measurements from Gunthe *et al.* (3), subsequently abbreviated as BEI. The data sets from the (so far unpublished) Delhi-2018 and EUREC^{4}A campaign were added here as well and κ_{org} and κ_{inorg} were calculated based on bivariate fits of the individual data sets.

Campaign, Site, Country	Time period	Conditions	Size [nm]	κ_{org}	κ_{inorg}	Reference
FACE-2005, Feldberg, DEU	22 Jun 2005 – 06 Jul 2005	rural background	< 100	0.10	0.69	Dusek *et al.* (4)
MIRAGE-2006, Mexico City, MEX	16 Mar 2006 – 31 Mar 2006	suburban site, megacity	100	0.04	0.46	Lance *et al.* (5)
PRIDE-PRD2006, Guangzhou, CHN	01 Jun 2006 – 30 Jun 2006	rural site around megacity	25 – 160	0.19	0.64	Rose *et al.* (2)
CAREBeijing-2006, Yufa, CHN	10 Aug 2006 – 09 Sep 2006	suburban site, megacity	44 – 190	0.06	0.68	Gunthe *et al.* (3)
CLACE-6, Jungfraujoch, CHE	03 Mar 2007 – 13 Mar 2007	high-alpine	~ 170	0.18	0.63	Rose *et al.* (6)
AMAZE-08, ZF2, BRA	14 Feb 2008 – 12 Mar 2008	wet season, rain forest	~ 200	0.09	0.63	Gunthe *et al.* (7)
BEACHON project, Colorado, USA	Mar 2010 – May 2011 & Jul 2011 – Aug 2011	semi-arid forest	160 – 210	0.13	0.60	Levin *et al.* (8, 9)
ATTO, BRA	23 Mar 2014 – 12 Feb 2015	full year, rain forest	~ 170	0.10	0.71	Pöhlker *et al.* (1)
Delhi, IND	05 Feb 2018 – 02 Mar 2018	megacity, winter	~ 140	0.10	0.65	this study
EUREC^{4}A, BRB	21 Jan 2020 to 22 Feb 2020	marine background	~ 120	0.25	0.61	this study

Table S2. Results of the linear bivariate regression fits of ϵ_{org} vs. κ from campaigns PRD, BEI, ATTO, as well as a merged case combining of all these data sets as shown in Fig. 1B. M and b denote the slope and the intercept of the linear fit $\kappa = M \cdot \epsilon_{org} + b$; the corresponding correlation coefficients R^2, the number of data points n in the individual data sets, as well as the corresponding retrieval of κ_{org} and κ_{inorg}. Note that the retrieved κ_{org} and κ_{inorg} in this table were obtained with the approach as outlined in the experimental section and deviate from the retrievals in the original studies as summarized Table S1. The main differences are the binning on the ϵ_{org} axis and the application of the linear bivariate fit applied here.

Campaign	M	b	R^2	κ_{org}	κ_{inorg}	n
ATTO	-0.45 ± 0.01	0.59 ± 0.01	0.97	0.13 ± 0.02	0.59 ± 0.01	1503
PRD	-0.60 ± 0.02	0.66 ± 0.01	0.88	0.06 ± 0.03	0.66 ± 0.02	410
BEI	-0.61 ± 0.03	0.68 ± 0.02	0.90	0.07 ± 0.03	0.68 ± 0.02	261
**Merged**	**-0.51 ± 0.01**	**0.63 ± 0.01**	**0.97**	**0.12 ± 0.02**	**0.63 ± 0.01**	**2179**

Table S3. Same as Table 1 for "high OC" conditions with a organic carbon burden larger than 10 mg m^{-2} in monthly means. Uncertainty in RFari and ERFaer represents the standard deviation of the multi-year simulations.

Model experiment	κ_{inorg}	κ_{org}	RFari, high OC [W m^{-2}]	ERFaer, high OC [W m^{-2}]
Reference case	0.60	0.06	0.413 ± 0.023	−3.669 ± 0.074
$\kappa_{OC} = \kappa_{org}$	0.60	0.12	0.380 ± 0.022	−3.701 ± 0.074
$\kappa_{SU} = \kappa_{inorg}$	0.63	0.06	0.399 ± 0.023	−3.735 ± 0.074
$\kappa_{OC} = \kappa_{org}$ & $\kappa_{SU} = \kappa_{inorg}$	0.63	0.12	0.364 ± 0.022	−3.818 ± 0.075

Table S4. List of acronyms.

Acronym	Description
aci	aerosol-cloud interactions
ACSM	aerosol chemical speciation monitor
AMS	aerosol mass spectrometer
ari	aerosol-radiation interactions
ATTO	Amazon Tall Tower Observatory
BEI	CAREBeijing-2006 campaign in China
CCN	cloud condensation nuclei
ECHAM–HAM	ECHAM atmospheric general-circulation model coupled to the HAM module
ECHAM	general atmospheric circulation model developed by the Max Planck Institute for Meteorology in Hamburg
ERA	European Centre for Medium-Range Weather Forecasts (ECMWF) Re-Analysis
ERF	effective radiative forcing
RFaci	RF from aerosol–cloud interactions (without rapid adjustments)
RFari	RF from aerosol–radiation interactions
ERFaer	ERF from aerosol–radiation and aerosol–cloud interactions (with rapid adjustments)
HAM	Hamburg Aerosol Model
PD	present day
PI	pre-industrial
PRD	PRIDE-PRD2006 campaign in China
RF	radiative forcing
SIC	sea ice cover
SST	sea surface temperature
ZSR	Zdanovskii, Stokes, and Robinson

Table S5. List of symbols.

Description	Symbol	Unit
Ammonium	NH_4^+	
Chloride	Cl^-	
Density	ρ	g cm^{-3}
Dry component volume fraction	ϵ_i	
Hygroscopicity parameter	κ	–
Hygroscopicity parameter of organic aerosol fraction	κ_{org}	–
Hygroscopicity parameter of organic aerosol used in ECHAM–HAM	κ_{OC}	–
Hygroscopicity parameter of inorganic aerosol fraction	κ_{inorg}	–
Hygroscopicity parameter of sulfate aerosol used in ECHAM–HAM	κ_{SU}	–
Hygroscopicity parameter of sea salt used in ECHAM–HAM	κ_{SS}	–
Hygroscopicity parameter of mineral dust used in ECHAM–HAM	κ_{DU}	–
Hygroscopicity parameter of black carbon used in ECHAM–HAM	κ_{BC}	–
Inorganic mass fraction	ϵ_{inorg}	
Mass concentration of Org	m_{org}	µg m^{-3}
Mass concentration of SO_4^{2-}	$m_{SO_4^{2-}}$	µg m^{-3}
Mass concentration of NO_3^-	$m_{NO_3^-}$	µg m^{-3}
Mass concentration of NH_4^+	$m_{NH_4^+}$	µg m^{-3}
Mass concentration of Cl^-	m_{Cl^-}	µg m^{-3}
Nitrate	NO_3^-	
Organics	Org	
Organic mass fraction	ϵ_{org}	–
Sensitivity	ξ	
Sulfate	SO_4^{2-}	
Total aerosol mass	m_{total}	
Volume of individual aerosol components	V_{si}	
Water activity	a_w	–
Water vapor saturation	s	–
Water vapor supersaturation	S	%

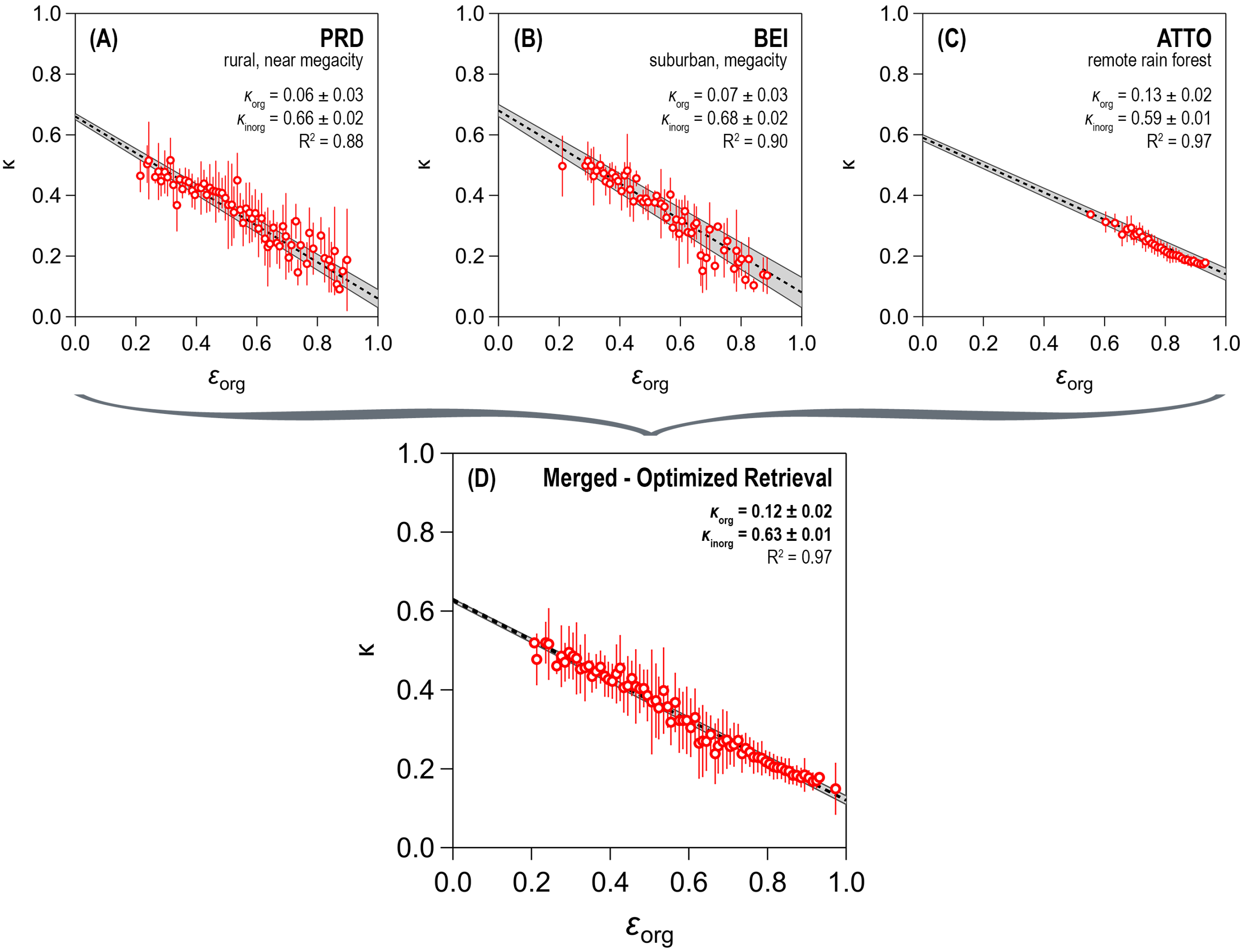

Figure S1. Retrieval of κ_{org} and κ_{inorg} based on three field data sets: (A) PRIDE-PRD2006 measurements (PRD) (2), (B) CAREBeijing-2006 measurements (BEI) (3), (C) a full year measurement at the Amazon Tall Tower Observatory (ATTO) (1) as well as (D) a merged case with all these data being combined. Linear regression fits and retrieved κ_{org} and κ_{inorg} are somewhat variable among individual campaigns (A-C). The merged case with a combination of data sets from contrasting environments (rain forest vs. megacity) in (D) shows a remarkably tight linear correlation. The fit parameters of the regression fits, correlation coefficients R^2, and retrieved κ_{org} and κ_{inorg} values are summarized in in each panel and in Table S2. Markers represent geometric mean of ϵ_{org} bins and error bars standard deviation. The gray shading of the regression fits represents the uncertainty of the fit.

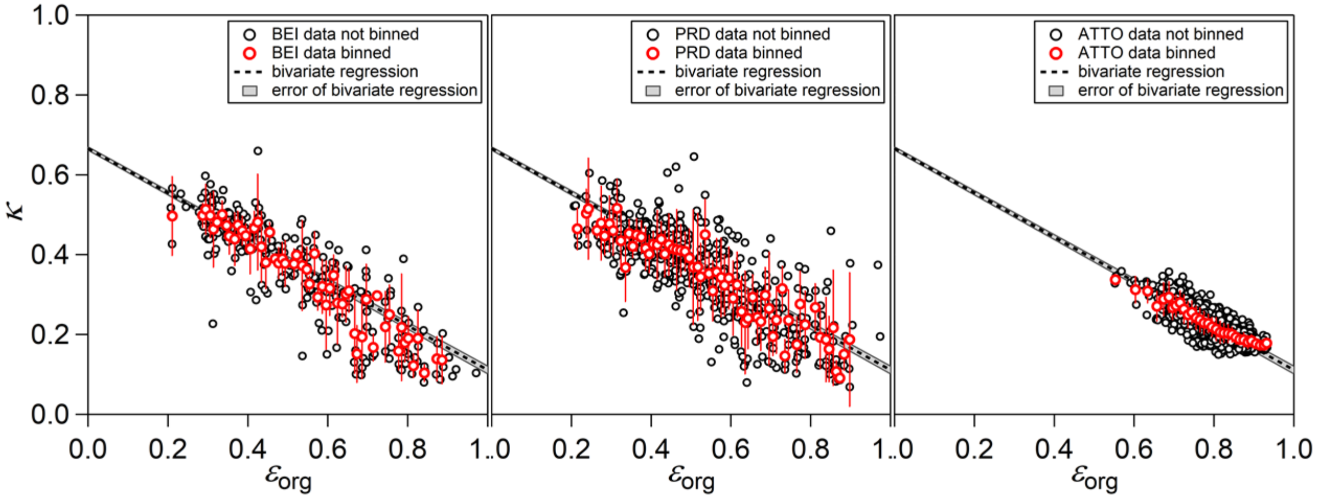

Figure S2. Field data sets PRD, BEI, and ATTO (see Fig. S1) plotted with and without binning of κ onto an ϵ_{org} grid increments of 0.01. The dashed lines represent the regression fit from Fig. S1D. Error bars at red markers represent one standard deviation.

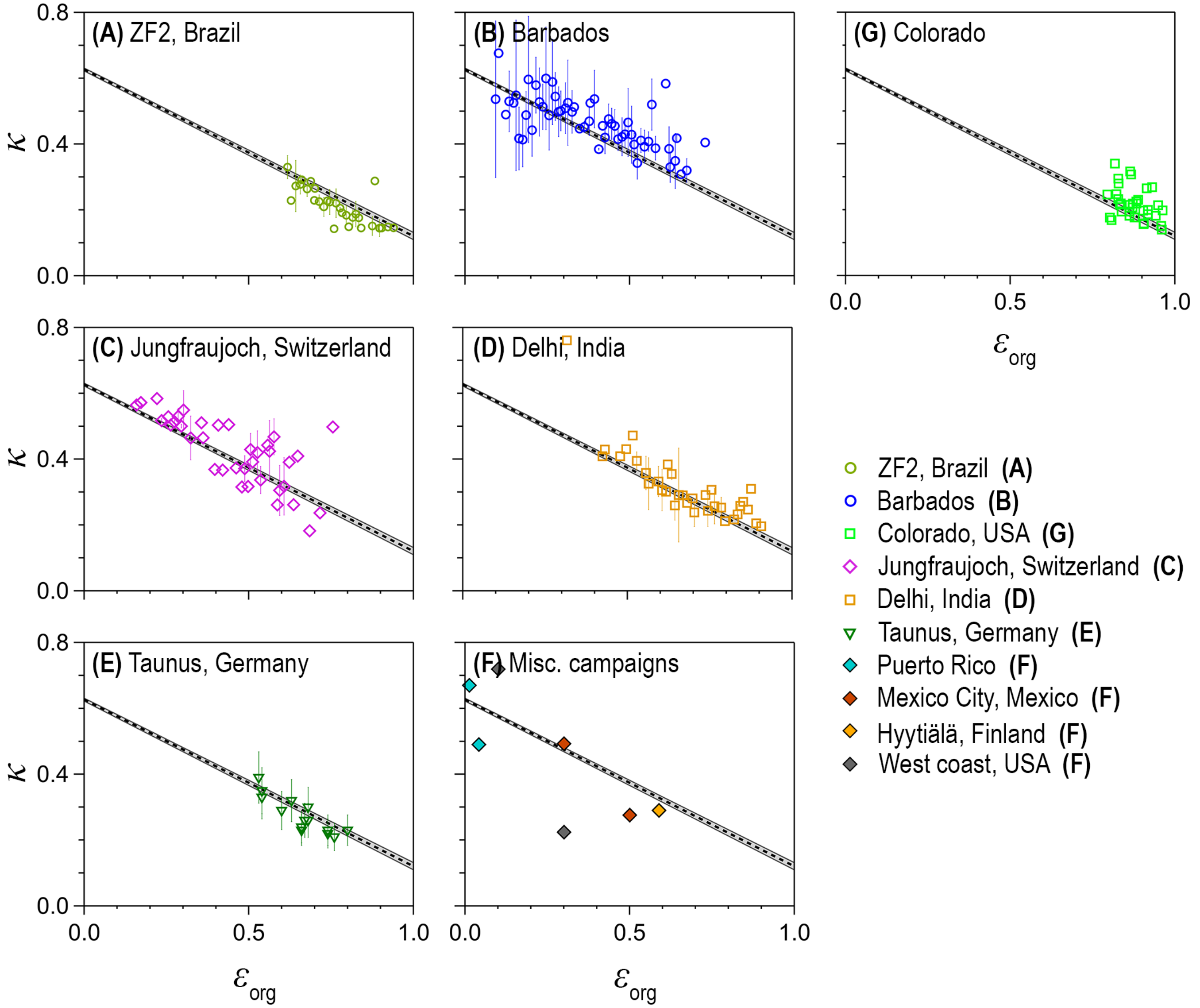

Figure S3. Separate representation of all field data sets that are shown in combination in Fig. 1C. Note that colors and shapes of the markers here and in Fig. 1A and C are identical to related the data sets to the corresponding sites. Markers represent geometric mean of ϵ_{org} bins and error bars standard deviation.

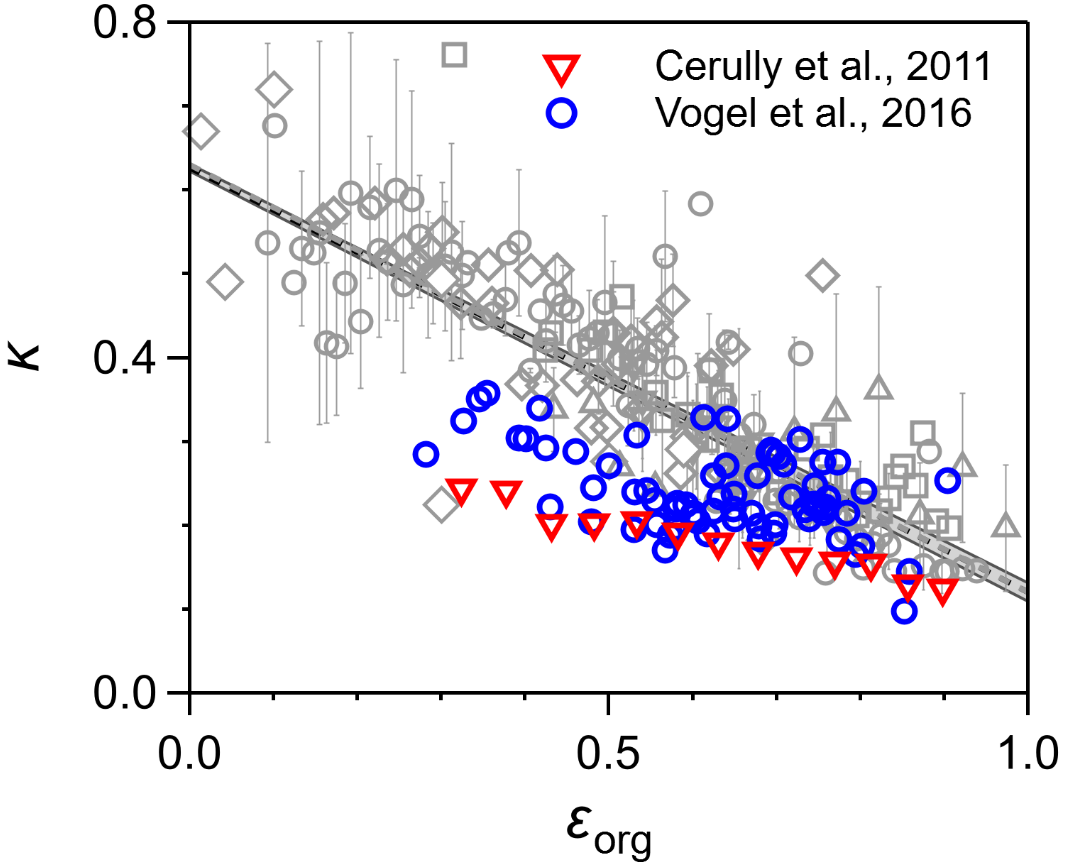

Figure S4. All campaign data sets from Fig. 1C shown here in gray, in combination with data from Vogel *et al.* (10) in blue and Cerully *et al.* (11) in red. These data sets are the only cases that deviate from the overall linear relationship. In fact, both studies also provide plausible explanations for the deviations: Vogel *et al.* (10) reported a clear deviation to lower κ values for rather fresh aerosols, especially when high fractions of organic bonded sulfate were involved, whereas more aged aerosols agree well with the common linear trend line. Cerully *et al.* (11) combined aerosol mass spectrometer and CCN measurements in different size ranges, which likely causes the underestimation of κ_{inorg} here.

Supplementary References

[1] M. L. Pöhlker, C. Pöhlker, T. Klimach, I. Hrabe de Angelis, H. M. J. Barbosa, J. Brito, S. Carbone, Y. Cheng, X. Chi, F. Ditas, R. Ditz, S. S. Gunthe, J. Kesselmeier, T. Könemann, J. V. Lavrič, S. T. Martin, D. Moran-Zuloaga, D. Rose, J. Saturno, H. Su, R. Thalman, D. Walter, J. Wang, S. Wolff, P. Artaxo, M. O. Andreae, U. Pöschl, Long-term observations of cloud condensation nuclei in the Amazon rain forest – Part 1: Aerosol size distribution, hygroscopicity, and new model parameterizations for CCN prediction, *Atmospheric Chemistry and Physics* **16**, 15709–15740 (2016).

[2] D. Rose, S. S. Gunthe, H. Su, R. M. Garland, H. Yang, M. Berghof, Y. F. Cheng, B. Wehner, P. Achtert, A. Nowak, A. Wiedensohler, N. Takegawa, Y. Kondo, M. Hu, Y. Zhang, M. O. Andreae, U. Pöschl, Cloud condensation nuclei in polluted air and biomass burning smoke near the mega-city Guangzhou, China – Part 2: Size-resolved aerosol chemical composition, diurnal cycles, and externally mixed weakly CCN-active soot particles, *Atmospheric Chemistry and Physics* **11**, 2817–2836 (2011).

[3] S. S. Gunthe, D. Rose, H. Su, R. M. Garland, P. Achtert, A. Nowak, A. Wiedensohler, M. Kuwata, N. Takegawa, Y. Kondo, M. Hu, M. Shao, T. Zhu, M. O. Andreae, U. Poeschl, Cloud condensation nuclei (CCN) from fresh and aged air pollution in the megacity region of Beijing, *Atmospheric Chemistry and Physics* **11**, 11023–11039 (2011).

[4] U. Dusek, G. P. Frank, J. Curtius, F. Drewnick, J. Schneider, A. Kürten, D. Rose, M. O. Andreae, S. Borrmann, U. Pöschl, Enhanced organic mass fraction and decreased hygroscopicity of cloud condensation nuclei (CCN) during new particle formation events, *Geophysical Research Letters* **37** (2010).

[5] S. Lance, T. Raatikainen, T. B. Onasch, D. R. Worsnop, X.-Y. Yu, M. L. Alexander, M. R. Stolzenburg, P. H. McMurry, J. N. Smith, A. Nenes, Aerosol mixing state, hygroscopic growth and cloud activation efficiency during MIRAGE 2006, *Atmospheric Chemistry and Physics* **13**, 5049–5062 (2013).

[6] D. Rose, S. S. Gunthe, Z. Jurányi, M. Gysel, G. P. Frank, J. Schneider, J. Curtius, U. Pöschl, Size-resolved and integral measurements of cloud condensation nuclei (CCN) at the high-alpine site Jungfraujoch, *Atmospheric Chemistry and Physics Discussions* **2013**, 32575–32624 (2013).

[7] S. S. Gunthe, S. M. King, D. Rose, Q. Chen, P. Roldin, D. K. Farmer, J. L. Jimenez, P. Artaxo, M. O. Andreae, S. T. Martin, U. Poschl, Cloud condensation nuclei in pristine tropical rainforest air of Amazonia: size-resolved measurements and modeling of atmospheric aerosol composition and CCN activity, *Atmospheric Chemistry and Physics* **9**, 7551–7575 (2009).

[8] E. J. T. Levin, A. J. Prenni, M. D. Petters, S. M. Kreidenweis, R. C. Sullivan, S. A. Atwood, J. Ortega, P. J. DeMott, J. N. Smith, An annual cycle of size-resolved aerosol hygroscopicity at a forested site in colorado, *Journal of Geophysical Research: Atmospheres* **117**, 1–13 (2012).

[9] E. J. T. Levin, A. J. Prenni, B. B. Palm, D. A. Day, P. Campuzano-Jost, P. M. Winkler, S. M. Kreidenweis, P. J. DeMott, J. L. Jimenez, J. N. Smith, Size-resolved aerosol composition and its link to hygroscopicity at a forested site in colorado, *Atmospheric Chemistry and Physics* **14**, 2657–2667 (2014).

[10] A. L. Vogel, J. Schneider, C. Muller-Tautges, G. J. Phillips, M. L. Pohlker, D. Rose, C. Zuth, U. Makkonen, H. Hakola, J. N. Crowley, M. O. Andreae, U. Poschl, T. Hoffmann, Aerosol chemistry resolved by mass spectrometry: Linking field measurements of cloud condensation nuclei activity to organic aerosol composition, *Environmental Science & Technology* **50**, 10823–10832 (2016).

[11] K. M. Cerully, T. Raatikainen, S. Lance, D. Tkacik, P. Tiitta, T. Petäjä, M. Ehn, M. Kulmala, D. R. Worsnop, A. Laaksonen, J. N. Smith, A. Nenes, Aerosol hygroscopicity and CCN activation kinetics in a boreal forest environment during the 2007 EUCAARI campaign, *Atmospheric Chemistry and Physics* **11**, 12369–12386 (2011).
